# Entrepreneurship and the Racial Wealth Gap: The Impact of Entrepreneurial Success or Failure on the Wealth Mobility of Black and White Families

**DOI:** 10.1007/s41996-021-00081-6

**Published:** 2021-02-22

**Authors:** Teresa Kroeger, Graham Wright

**Affiliations:** Brandeis University, Waltham, MA USA

**Keywords:** Entrepreneurship;, Racial wealth gap;, Economic mobility;, United States

## Abstract

Research has repeatedly argued that increasing the rate at which Black people start businesses could reduce the racial wealth gap between Black and white families, but increasing the rate of Black entrepreneurship may actually exacerbate the racial wealth gap, due to the economic cost associated with business closure. Using longitudinal data from the Panel Study of Income Dynamics (PSID), we find that, as past work suggests, Black-owned businesses are less likely to remain open 4 years later, compared to white-owned businesses, and that, due to this disparity, Black business owners are more likely to experience downward economic mobility and less likely to experience upward mobility, compared to their white counterparts. These results suggest that improving the rate at which Black entrepreneurs succeed, rather than increasing the rate at which Black people become entrepreneurs, should be the target of efforts to leverage business ownership to reduce the racial wealth gap.

**Supplementary Information:**

The online version contains supplementary material available at 10.1007/s41996-021-00081-6.

## Introduction

Research has repeatedly argued that increasing the rate at which Black people start businesses could reduce the racial wealth gap between Black and white families (Boston, 1999; Bradford, 2014; Butler, 1991). However, past scholarship has not sufficiently accounted for the possibility that increasing the rate of Black entrepreneurship may actually exacerbate the racial wealth gap, due to the economic cost associated with business closure. Understanding the economic consequences of business failure is especially important in light of the large-scale economic disruptions, especially among Black-owned small businesses, that occurred during the COVID-19 pandemic (Fairlie, 2020). In this paper, we analyze longitudinal data from the Panel Study of Income Dynamics (PSID) to illustrate both the opportunities and losses that business success or failure offers for Black and white entrepreneurs.

We first find that, as past work suggests, Black-owned businesses are less likely to remain open 4 years later, compared to white-owned businesses. We then show that Black and white entrepreneurs have a similar likelihood of experiencing upward economic mobility when their businesses succeed, and similar likelihoods of experiencing downward mobility when their businesses fail. Finally, as predicted by these two findings, Black business owners in a given year are more likely to experience downward economic mobility over the next 4 years, and less likely to experience upward mobility, compared to their white counterparts. In light of these results, improving the rate at which Black entrepreneurs succeed, rather than increasing the rate at which Black people become entrepreneurs, should be the primary focus of efforts to leverage business ownership to reduce the racial wealth gap.

## Background

There is much evidence of the persistent and growing “wealth gap” between Black and white families in America (Gittleman & Wolff, 2004; Oliver & Shapiro, 2004; Shapiro et al., 2013). As scholars have begun to highlight the centrality of wealth, as opposed to income, in the challenge of economic inequality (Piketty, 2014; Sherraden, 1991), substantial effort has been devoted to identifying policies that might reduce this “racial wealth gap.” Some popularly debated policies include tax credits to help first time homebuyers afford a down payment (Shapiro, 2004), “baby bonds” (Hamilton & Darity Jr., 2010), and elimination of the mortgage interest tax credit (Sullivan et al., 2017). At the same time, research also shows that some policies aimed at reducing economic inequality, such as eliminating student debt for all Americans, would actually *exacerbate* the racial wealth gap (Sullivan et al., 2015). The full economic implications of a policy are not always obvious at first glance.

For decades, researchers and advocates have pointed to entrepreneurship as a mechanism for reducing economic disparities between Black and white households. This work argues that promoting entrepreneurship and business formation within the Black community will lead to increased opportunities for upward economic mobility, and greater integration into the American economy (Boston, 1999; Butler, 1991). In the abstract, this idea has merit, since research has found that entrepreneurs experience greater upward wealth mobility compared to workers (Quadrini, 1999). Nevertheless, the theory that entrepreneurship could help reduce the racial wealth gap has rarely been tested empirically.^1^

One of the few recent attempts to directly investigate the relationship between entrepreneurship and the racial wealth gap was by Bradford (2014). Using data from the Panel Study of Income Dynamics (PSID), Bradford studied the impact of entrepreneurship on the wealth mobility of Black and white families over two 4-year periods: 2001–2005 and 2005–2009. After controlling for pre-existing differences in education, family composition, homeownership, and other salient factors, Bradford finds that:

The upward wealth mobility of Black entrepreneurs is also equivalent to that of White entrepreneurs, while the wealth mobility of White entrepreneurs is greater than that of White workers. These relationships are consistent with the existence of Black entrepreneurs reducing the wealth gap between Black and White families. (p. 267)

Consequently, Bradford argues that, as long as new Black entrepreneurs achieve the same economic benefits as those in his sample, “increasing the rate of Black entrepreneurship will reduce the wealth disparity between Black and White families” (p. 255). Especially in combination with earlier work on the benefits of entrepreneurship, Bradford’s results certainly offer cause for optimism about the role of entrepreneurship in reducing the racial wealth gap. At the same time, there are many reasons to be cautious about the claim that simply *increasing* Black entrepreneurship will lead to beneficial economic outcomes.

### Business Success and Failure

Starting a business is an inherently risky activity. Although the popular myth that “nine out of ten businesses fail in their first year” is an exaggeration (Phillips & Kirchhoff, 1989), Headd (2003) finds that around a third of new firms fail after 4 years. Business failure obviously has costs. These include financial costs, including high levels of personal debt that may hold back wealth creation for years (Cope, 2011), as well as psychological and social costs that may harm well-being in other ways (Ucbasaran et al., 2013). It is intuitively obvious that, while starting a *successful* business may lead to upward wealth mobility, starting a business that *fails* may lead to *downward* wealth mobility. This fact has clear but thus far unappreciated implications for debates over the relationship between entrepreneurship and the racial wealth gap, because it is well documented that Black and minority-owned businesses fail at higher rates than those owned by their white peers.

Researchers have long been aware of an apparent anomaly in regard to Black entrepreneurship. Although Black people appear to start new businesses at higher rates than white people (Köllinger & Minniti, 2006), they are less likely to remain business owners over their life course (Ahn, 2011). Subsequent research has confirmed what these two findings imply: businesses owned by minorities tend to be less successful than those owned by white people (Bates, 1989; Fairlie & Robb, 2008; Headd, 2003). The causes of this disparity have been the subject of much investigation. Fairlie and Robb (2008) flag a number of issues driving lower success rates among Black entrepreneurs, including a lack of familial experience in business ownership, but see the biggest contributor as a greater difficulty in acquiring startup capital. A robust body of research confirms that Black entrepreneurs continue to face severe racial discrimination in attempting to secure financing for their business, making the prospect of starting a successful business even harder than it is for their white counterparts. Statistical analyses find that race remains a negative predictor of loan acceptance and loan quality, even after controlling for salient economic characteristics (Asiedu et al., 2012; Blanchard et al., 2008; Blanchflower et al., 2003). Other work has confirmed that these disparities are not merely due to location-based “redlining” but are primarily a function of the entrepreneur’s own race (Bates & Robb, 2015, 2016). This discrimination can be clearly observed in “mystery shopper” studies that document severe disparities in the way bank employees treat equally qualified Black and white entrepreneurs asking for identical loans (Bone et al., 2014, 2017; Lubin, 2011; Turner et al., 2002). This strand of research has troubling implications for attempts to reduce the racial wealth gap by promoting Black entrepreneurship.

It may be true that successful Black entrepreneurs achieve upward wealth mobility at the same rate as white entrepreneurs. However, if Black entrepreneurs are more likely to see their businesses fail, and business failure leads to downward wealth mobility, then it is eminently plausible that increasing the rates at which Black people start businesses might exacerbate the racial wealth gap. Furthermore, insofar as Black entrepreneurs are offered credit on worse terms (e.g., higher interest rates) than white entrepreneurs (Fairlie & Robb, 2008), it is possible that the economic consequences of business failure may be more severe for Black entrepreneurs.

In this paper, we use the PSID to extend earlier work and study the implications of entrepreneurship on the racial wealth gap. We expand upon Bradford’s earlier approach in a number of ways. Firstly, we include new PSID data from 2009-2017, which substantially increases our statistical power. Secondly, we analyze racial disparities in both entrepreneurial success and failure, and explicitly test for racial disparities in the *impact* (vis a vis wealth mobility) of success and failure. Finally, we make use of new measures of entrepreneurship and wealth that more clearly correspond to the constructs in question. Using this paradigm, we explore the following research questions. Firstly, we examine whether the racial disparities in business success documented by previous literature are evident in the PSID data from 2001 to 2017. Secondly, we separately analyze the impact of successful and unsuccessful entrepreneurship on upward and downward wealth mobility, and test whether the magnitude of these impacts differs by race. Finally, in light of the results above, we analyze the net impact of *becoming* an entrepreneur on wealth mobility for Black and white families.

## Data and Methods

Using data from the PSID, we analyze how a family’s wealth and employment status change over a 4-year period. Because our focus is on the disparity between Black and white families, we limit our analyses to families where the PSID denoted “reference person”^2^ of the family is either Black non-Hispanic or white non-Hispanic. The central limitation in this analysis is the relatively small number of Black entrepreneurs that exist in any given year of the PSID. Following Bradford (2014) we mitigate this issue by combining four distinct “cohorts” of PSID respondents, with each cohort representing a different 4-year period. In addition to the 2001–2005, and 2005–2009 cohorts used in Bradford’s original analysis, we add two more: 2009–2013, and 2013–2017. Within each of these cohorts, we look at how wealth and employment status has changed between the first and second time point.^3^ As a consequence of this approach, the same family may exist in multiple cohorts (e.g., if they have data for 2001, 2005, 2009, and 2013), and as such robust standard errors are used to account for the clustering of observations within families. This approach provides us with a dataset of 22,958 observations across the four cohorts (see Table 1). All analyses are run using PSID calculated weights.

**Table 1:** Number of families in combined periods 2001–2005, 2005–2009, 2009–2013, and 2013–2017

	All families	White families	Black families
Period			
2001–2005	5117	3420	1697
2005–2009	5692	3648	2044
2009–2013	6006	3668	2338
2013–2017	6143	3535	2608
Total	22,958	14,271	8,687

Data in this table are unweighted to show the number of observations in the sample. Estimated using PSID data (2020)

### Variables

In analyzing the relationship between entrepreneurship and wealth, we operationalize these two concepts in a slightly different way from earlier work (e.g., Bradford, 2014), to more accurately reflect the underlying constructs. Firstly, while earlier work tended to analyze changes in total wealth, we limit our analyses to wealth excluding home equity.^4^ This decision helps to disentangle the economic effects of entrepreneurship with the dramatic changes in housing prices that occurred before, during, and after the 2009 housing crash and Great Recession. Of course, business ownership can also impact home equity in various ways, but in the time frame we analyze, ignoring these effects seems preferable to potentially confounding the wealth effects of entrepreneurship with dramatic swings in home prices that have little to do with a family’s personal financial situation.^5^

Secondly, past work operationalized entrepreneurship as “self-employment.” That is, individuals were considered to be entrepreneurs if they reported that they were working partly or entirely for themselves, as opposed to working for someone else (or not working for any reason). This definition does not, however, align particularly well with the way in which entrepreneurship is usually conceptualized by researchers, policymakers and the public at large, where it is usually treated as a synonym for *business ownership.*^6^ Use of self-employment as a proxy for entrepreneurship may be especially problematic given the rise of the “gig economy,” since independent contractors (e.g., drivers for ride-sharing services) may classify themselves as “self-employed,” despite not being “entrepreneurs” by any reasonable definition. Indeed, Abraham et al. (2018) find that about half of their respondents who said they were primarily self-employed classified themselves as independent contractors, independent consultants, or freelance workers. To avoid this issue, we define entrepreneurs as those respondents who indicated that the reference person owned a business or had financial interest in a business enterprise in the previous year.

We then define entrepreneurial “success” and “failure” by examining changes in business ownership and employment status over the 4 years between the two time points in each cohort. Individuals who were entrepreneurs in the first time point and were still entrepreneurs 4 years later are considered “successful entrepreneurs.” Unsuccessful entrepreneurs are those who owned a business in the first time point and did not own a business, but were either working or unemployed in the second time point.^7^ Individuals who were workers at both time points are considered “stayed workers” and are treated as a control group in assessing the impact of entrepreneurship in general.^8^ We therefore define a reference person’s “transition status” as whether, over the course of 4 years, they were successful entrepreneurs, unsuccessful entrepreneurs, or stayed workers. This variable is used as the key independent variable in models estimating the economic impact of entrepreneurial success or failure. To determine whether there are racial disparities in entrepreneurial success, we use a binary logit model to predict the likelihood that entrepreneurs at the first time point were “successful entrepreneurs” 4 years later, controlling for the race of the family’s reference person and other potential confounding variables, as discussed below.

Following earlier analyses, we define economic mobility as change in wealth tercile over the 4-year period in question. Families are assigned to a position in the top, middle, or bottom tercile with respect to net, non-equity wealth relative to the entire sample at each time point. Binary logistic regression models are used to predict the probability of a family rising into the top tercile from below the top, or falling into the bottom tercile from above the bottom, as a function of entrepreneurship status and other factors. Because wealth terciles are calculated separately for every year, the dependent variable for these analyses represents *relative*, rather than absolute, wealth mobility.^9^

We estimate the impact of entrepreneurship on wealth mobility using two different types of binary logit models. The first type of model predicts the likelihood of a family rising into the top wealth tercile from below the top, thereby estimating *upward* wealth mobility. The second type of model predicts the likelihood of a family falling into the bottom tercile from above the bottom, thereby estimating *downward* wealth mobility. We first deploy these models to determine the impact of transition status (i.e., being a successful or unsuccessful entrepreneur, vs being a worker), and again to determine the impact of being an entrepreneur in the first time point (as opposed to being a worker). In these models, we interact transition status or entrepreneurship with race to estimate whether any of the effects of entrepreneurship are significantly different for Black and white families.

In addition to entrepreneurship status and race, all models control for a number of potential confounding variables. These include education, age, household type (married, single male reference person, single female reference person), number of children, home ownership, health status, reception of gift or inheritance over $10,000 in the past 5 years, initial wealth tercile, and cohort. Table 2 shows descriptive statistics for all key variables included in these models, separately by the race of the reference person.

**Table 2:** Family traits in combined periods 2001–2005, 2005–2009, 2009–2013, and 2013–2017

	All families	White families	Black families	
Status at start				
Entrepreneur	0.099	0.111	0.035	***
Worker	0.576	0.575	0.584	
Retired	0.203	0.216	0.136	***
Unemployed	0.122	0.098	0.245	***
Education				
Less than high school	0.128	0.110	0.221	***
High school	0.299	0.289	0.350	***
Some college	0.251	0.245	0.281	***
Bachelor's degree	0.185	0.204	0.087	***
More than bachelor’s degree	0.138	0.153	0.060	***
Age (in years)				
Under 35	0.205	0.194	0.261	***
35 to 54	0.397	0.385	0.460	***
Over 54	0.398	0.421	0.280	***
Household type				
Single male	0.220	0.208	0.279	***
Single female	0.285	0.245	0.493	***
Married couple	0.496	0.547	0.227	***
Average no. of children under 18 years old	0.521	0.492	0.671	***
Standard deviation	0.963	0.930	1.108	
Own home	0.662	0.712	0.402	***
Health good or better	0.846	0.863	0.756	***
Received gift or inheritance	0.095	0.108	0.027	***
Middle third wealth at start	0.294	0.282	0.355	***
Top two thirds at start	0.739	0.784	0.504	***
Top two thirds at end	0.738	0.781	0.511	
Average wealth at start ($)	254,834	293,441	53,157	☨
Standard deviation	1,609,155	1,744,810	405,707	
Average wealth at end ($)	304,843	352,984	53,360	***
Standard deviation	1,801,321	1,954,229	412,466	
*N*	22,958	14,271	8,687	

Significance values from design-adjusted chi-square and *t* tests. Wealth is in nominal dollars. Estimated using PSID data (2020)

☨*p*<0.1, *p<0.05, **p<0.01, ***p<0.001

Although the small number of entrepreneurs (especially Black entrepreneurs) in the PSID make finer grained mobility analyses (e.g., using deciles or quartiles) infeasible, the limitations of this approach should nevertheless be acknowledged. Firstly, our approach can only detect fairly large changes in wealth and is blind to changes that may be economically consequential, but which are not large enough to change a family’s tercile. Secondly, by mathematical necessity, families who start at either the top or bottom tercile must be excluded from analyses of upward or downward mobility, respectively. This has particular implications for our ability to detect downward economic mobility as a result of unsuccessful entrepreneurship. Because entrepreneurs who start at the lowest tercile cannot, by definition, fall any further, they must be excluded from analyses of downward mobility. This implies that our analysis will likely underestimate the true impact of entrepreneurship on downward wealth mobility. In our data approximately 25% of Black entrepreneurs were in the lowest tercile at time 1 (versus 10% of white entrepreneurs). The exclusion of these observations further limits our power to detect downward mobility among Black entrepreneurs. Finally, it should be noted that an association between transition status and economic mobility does not definitively indicate the direction of the causal relationship. In particular, for many entrepreneurs, an exogenous shock to wealth may have led to business failure, as opposed to business failure leading to a decline in wealth. These methods can nonetheless shed light on the association between large shocks to wealth and entrepreneurship.

## Results

We first analyze racial disparities in business success over a 4-year period. Table 3 shows that, after controlling for other socioeconomic factors, Black reference persons who are entrepreneurs at a given time point are significantly less likely to still be entrepreneurs 4 years later, compared to white reference persons. This is in line with past work and suggests that, whatever the benefits of successful entrepreneurship are, Black entrepreneurs are less likely to receive those benefits, compared to white entrepreneurs. In contrast, Black entrepreneurs are more likely to experience whatever penalties are associated with unsuccessful entrepreneurship.

**Table 3:** Logit regression predicting successful entrepreneurship. Dependent variable: entrepreneur at the start and end of the period

	Model 1		
	Coefficient	*Z* score	
Black reference person	-0.8112	-3.0	**
Education			
Less than high school	-0.3006	-1.1	
Some college	-0.1793	-1.0	
Bachelor's degree	-0.0845	-0.5	
More than bachelor's degree	0.2152	1.0	
Age (in years)			
Under 35	-0.2741	-1.6	
Over 54	-0.0423	-0.3	
Household type			
Single male	0.1104	0.5	
Single female	-0.1232	-0.5	
Number of children under 18	-0.1172	-1.9	☨
Own home	0.2359	1.2	
Health good or better	0.4311	1.7	☨
Received gift or inheritance	-0.0978	-0.5	
Wealth tercile			
Bottom third wealth at start	-0.9915	-5.1	***
Middle third wealth at start	-0.6278	-3.7	***
Period			
2001–2005	-0.1172	-0.7	
2005–2009	-0.0353	-0.2	
2009–2013	-0.0504	-0.3	
Constant	0.5938	1.7	☨

*N*=1,810. Regression includes only entrepreneurs at the start of the period. Robust standard errors clustered by family are used. Reference categories are, respectively, white reference person, high school degree, ages 35 to 54, married, no children under 18, does not own home, health worse than “good,” did not receive gift or inheritance, top or bottom third wealth at start, and 2013–2017. Transitions are measured across 4-year periods from 2001 to 2017. Estimated using PSID data (2020)

☨*p*<0.1, *p<0.05, **p<0.01, ***p<0.001

We now estimate the economic effects of successful and unsuccessful entrepreneurship, and test for the existence of racial disparities with respect to these effects. For each analysis, we present two logit models, one without any interaction terms (Model 1) and a second interacting the race of the reference person with entrepreneurship status (Model 2). Table 4 shows these two models predicting upward wealth mobility—the probability that a family will move into the upper wealth tercile from below the top third. Model 1 shows that, as expected, *successful* entrepreneurs are significantly more likely to experience upward mobility compared to those who stayed workers. Regardless of their transition status, Black reference persons are less likely to experience upward mobility compared to white reference persons. In Model 2, however, the interaction terms between race and transition status are non-significant, indicating that the relationship between successful entrepreneurship and upward mobility is not significantly different for Black and white families.

**Table 4:** Logit regressions predicting family transitions in the wealth distribution based on employment transition. Dependent variable: family rises into the top third from below the top third

	Model 1			Model 2		
	Coefficient	*Z* score		Coefficient	*Z* score	
Employment transition						
Successful entrepreneur	0.7778	4.4	***	0.7448	4.0	***
Unsuccessful entrepreneur	-0.0922	-0.4		-0.0607	-0.3	
Otherª	0.0830	1.0		0.0987	1.1	
Black reference person	-0.3117	-3.2	**	-0.2761	-2.2	*
Employment transition × Race						
Black successful entrepreneur				0.6503	1.4	
Black unsuccessful entrepreneur				-0.3057	-0.6	
Black other				-0.0896	-0.5	
Education						
Less than high school	-0.4473	-3.5	***	-0.4448	-3.5	***
Some college	0.3272	3.6	***	0.3285	3.6	***
Bachelor's degree	0.5996	5.9	***	0.5991	5.9	***
More than bachelor's degree	0.9334	7.8	***	0.9317	7.8	***
Age (in years)						
Under 35	-0.2934	-3.7	***	-0.2933	-3.7	***
Over 54	-0.0806	-0.8		-0.0824	-0.8	
Household type						
Single male	-0.2790	-2.9	**	-0.2794	-2.9	**
Single female	-0.6953	-7.0	***	-0.6953	-7.0	***
Number of children under 18	-0.1384	-3.9	***	-0.1388	-4.0	***
Own home	0.7991	9.3	***	0.8001	9.3	***
Health good or better	0.2544	2.3	*	0.2544	2.3	*
Received gift or inheritance	0.6758	5.4	***	0.6765	5.4	***
Middle third wealth at start	0.8558	10.9	***	0.8562	10.9	***
Period						
2001–2005	-0.3320	-3.4	***	-0.3316	-3.4	***
2005–2009	-0.1265	-1.3		-0.1268	-1.3	
2009–2013	-0.1456	-1.6		-0.1445	-1.6	
Constant	-2.5964	-15.5	***	-2.6023	-15.4	***

*N*=14,683. Robust standard errors clustered by family are used. Reference categories are, respectively, stayed worker, white reference person, Black stayed worker, high school degree, ages 35 to 54, married, no children under 18, does not own home, health worse than “good,” did not receive gift or inheritance, top or bottom third wealth at start, and 2013–2017. Transitions are measured across 4-year periods from 2001 to 2017. Estimated using PSID data (2020)

ªExcludes stayed worker

☨*p*<0.1, *p<0.05, **p<0.01, ***p<0.001

**Fig. 1:**
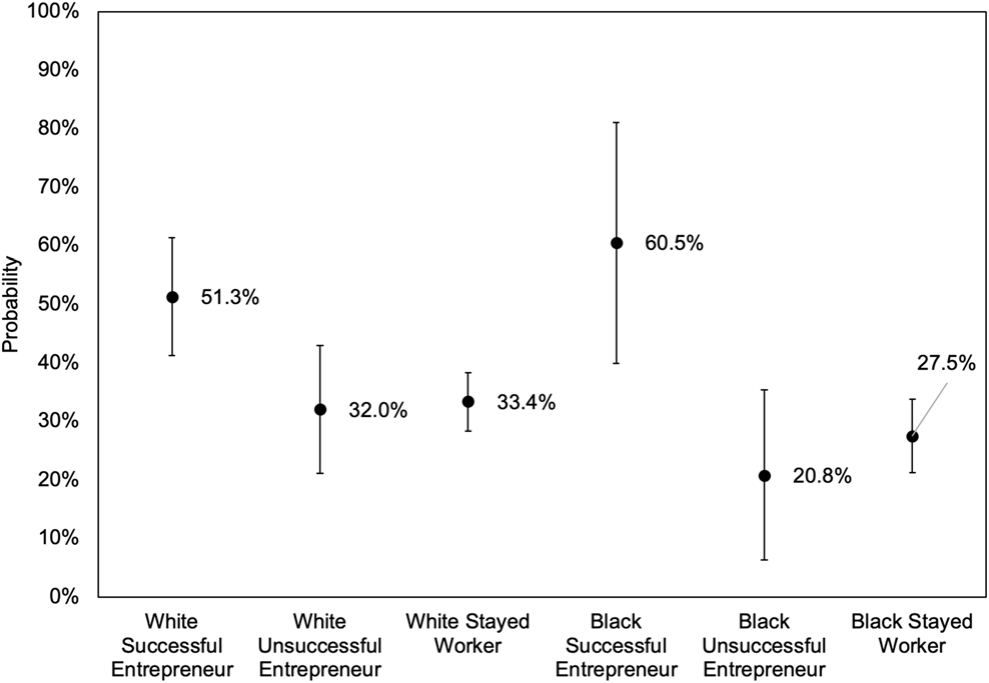
Predicted probability of rising into the top wealth tercile from below the top wealth tercile, by race and employment transition. *Note: Error bars represent 95% confidence intervals. Predictions derived from Model 2 in Table* *4*. *Other variables held at the following values: high school degree, age* *35 to 54, married, no children under 18, owns home, health better than “good,” did not receive gift or inheritance, middle third wealth at start, and 2013–2017. Transitions are measured across 4-year periods from 2001 to 2017*

To illustrate the magnitude of these effects, we use Model 2 to predict the probability of experiencing upward mobility for comparable Black and white families with different transition statuses. Figure 1 shows that, for both Black and white families, successful entrepreneurs are far more likely to experience upward mobility compared to either workers or unsuccessful entrepreneurs and this effect is at least as large, if not even larger, for Black families.

We now turn to the effects of transition status on downward wealth mobility, presented in Table 5. Model 1 shows that, compared to those who stayed workers, unsuccessful entrepreneurs and those with an “other” transition status (viz. worker to entrepreneur, unemployed to entrepreneur, worker to retired, or entrepreneur to retired) are significantly more likely to experience downward wealth mobility. Successful entrepreneurs are not significantly more or less likely to experience downward wealth mobility than those who stayed workers. After controlling for transition status and other factors, families with a Black reference person are also more likely to experience downward economic mobility compared to families with a white reference person. Once again, in Model 2, interaction terms between race and transition status are not significant, implying that the relationship between transition status and downward mobility are not significantly different for Black and white families.

**Table 5:** Logit regressions predicting family transitions in the wealth distribution based on employment transition. Dependent variable: family falls into the bottom third from above the bottom third

	Model 1			Model 2		
	Coefficient	*Z* score		Coefficient	*Z* score	
Employment transition						
Successful entrepreneur	-0.2607	-1.4		-0.3399	-1.8	☨
Unsuccessful entrepreneur	0.7463	4.3	***	0.6253	3.2	**
Otherª	0.1684	2.0	*	0.1230	1.3	
Black reference person	0.3366	3.7	***	0.2054	1.7	
Employment transition × Race						
Black successful entrepreneur				0.9135	1.4	
Black unsuccessful entrepreneur				0.7913	1.6	
Black other				0.2021	1.2	
Education						
Less than high school	0.2459	2.3	*	0.2440	2.3	*
Some college	-0.0941	-1.1		-0.0970	-1.1	
Bachelor’s degree	-0.4957	-4.6	***	-0.5008	-4.6	***
More than bachelor’s degree	-0.4085	-3.0	**	-0.4184	-3.1	**
Age (in years)						
Under 35	0.1909	2.2	*	0.1903	2.2	*
Over 54	-0.4736	-4.7	***	-0.4675	-4.6	***
Household type						
	0.2925	3.2	**	0.2884	3.1	**
Single female	0.7084	8.1	***	0.7109	8.1	***
Number of children under 18	0.1141	3.4	***	0.1115	3.4	***
Own home	-0.6303	-7.6	***	-0.6332	-7.6	***
Health good or better	-0.5210	-5.5	***	-0.5169	-5.5	***
Received gift or inheritance	-0.6045	-4.3	***	-0.6010	-4.3	***
Middle third wealth at start	1.2838	14.8	***	1.2804	14.8	***
Period						
2001–2005	0.0891	0.9		0.0895	0.9	
2005–2009	0.0978	1.0		0.0955	1.0	
2009–2013	-0.0595	-0.6		-0.0590	-0.6	
Constant	-1.9650	-11.5	***	-1.9337	-11.2	***

*N*=14,939. Robust standard errors clustered by family are used. Reference categories are, respectively, stayed worker, white reference person, Black stayed worker, high school degree, ages 35 to 54, married, no children under 18, does not own home, health worse than “good,” did not receive gift or inheritance, top or bottom third wealth at start, and 2013–2017. Transitions are measured across 4-year periods from 2001 to 2017. Estimated using PSID data (2020)

ªExcludes stayed worker

☨*p*<0.1, *p<0.05, **p<0.01, ***p<0.001

**Fig. 2:**
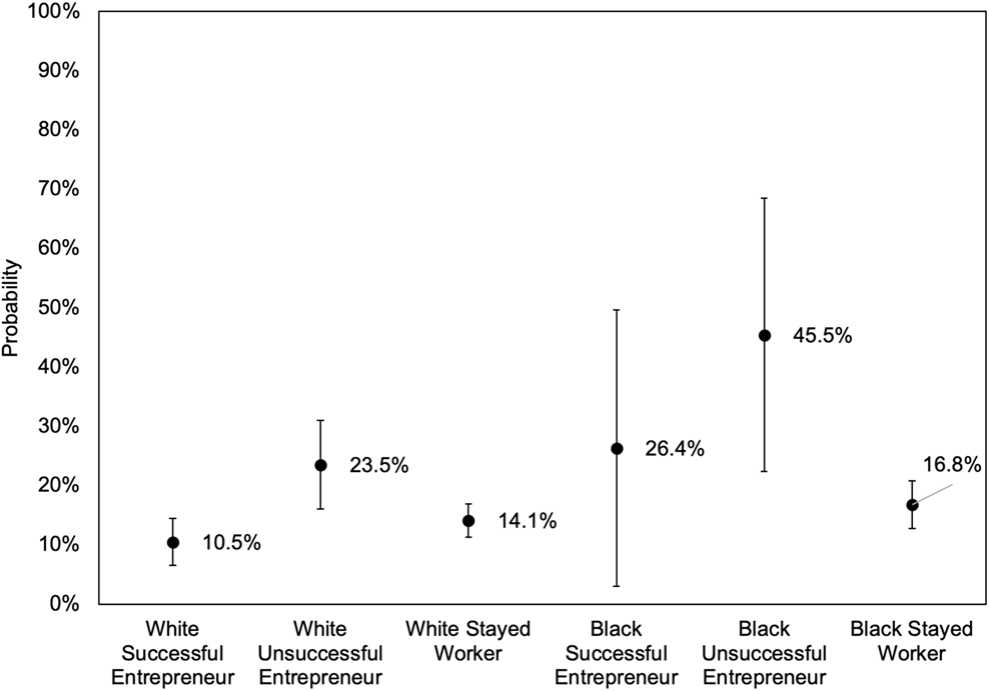
Predicted probability of falling into the bottom wealth tercile from above the bottom wealth tercile, by race and employment transition. *Note: Error bars represent 95% confidence intervals. Predictions derived from Model 2 in Table* *5**. Other variables held at the following values: high school degree, age 35 to 54, married, no children under 18, owns home, health better than* “*good,*” *did not receive gift or inheritance, middle third wealth at start, and 2013*–*2017. Transitions are measured across 4-year periods from 2001* *to* *2017*

As above, Fig. 2 presents predicted probabilities for experiencing downward wealth mobility derived from Model 2 of Table 5. For families with either a Black or white reference person, unsuccessful entrepreneurs are far more likely to experience downward economic mobility compared to successful entrepreneurs. However, although the interaction terms in Model 2 are not significant, it is clear that, from a substantive perspective, unsuccessful entrepreneurship poses a much more serious threat to Black entrepreneurs compared to their white counterparts. An unsuccessful Black entrepreneur who started in the middle wealth tercile has around a 50% chance of falling into the bottom tercile, while an unsuccessful white entrepreneur in a similar position has less than a 25% chance of falling into the bottom tercile (Fig. 2).

**Fig. 3:**
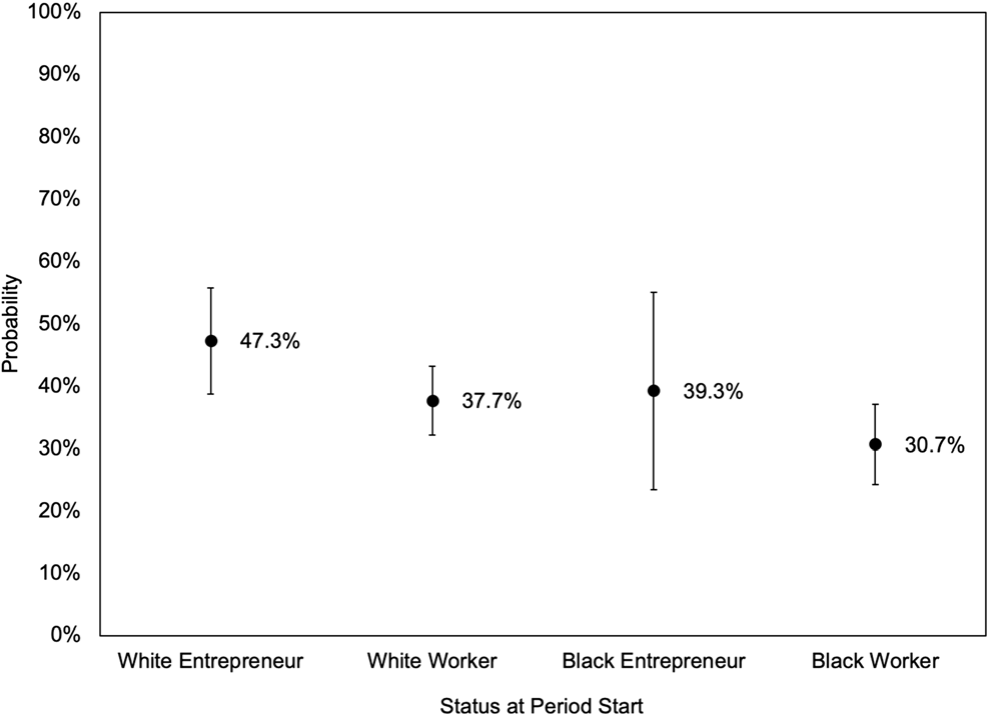
Predicted probability of rising into the top wealth tercile from below the top wealth tercile, by race and status at start. ***Note:*** *Error bars represent 95% confidence intervals. Predictions derived from Model 2 in Table* *6**. Other variables held at the following values: high school degree, age 35 to 54, married, no children under 18, owns home, health better than* “*good,*” *did not receive gift or inheritance, middle third wealth at start, and 2013–2017. Transitions are measured across 4-year periods from 2001* *to* *2017*

Although the confidence intervals around estimates for Black entrepreneurs are large, the above analyses suggest that, with respect to economic mobility, the economic payoffs for entrepreneurial success and failure are roughly similar for Black and white entrepreneurs. Yet the analysis in Table 5 finds that white entrepreneurs are more likely to receive the benefits of success, and less likely to pay the penalty of failure, compared to their Black counterparts. In combination, these two findings suggest that the decision to become an entrepreneur is a better “bet” for white people than for Black people. But how much better? To answer this question, and to estimate the impact of becoming an entrepreneur (i.e., starting a new venture without knowing whether it will succeed or fail), we specify new models estimating the relationship between wealth mobility and being an entrepreneur (versus a worker) at the first time point (time 1), regardless of entrepreneurship status at the end of the 4-year period (time 2). Table 6 shows the analysis with respect to upward economic mobility. Model 1 shows that reference persons who were entrepreneurs at time 1 were, overall, more likely to experience upward wealth mobility during the next 4 years. In Model 2, the interaction between race and entrepreneurship status is positive but non-significant, indicating that, despite their lower likelihood of success, Black people who were entrepreneurs at a given time point were not significantly less likely to experience upward mobility over the next 4 years. Figure 3 estimates predicted probabilities from Model 2 to estimate the overall likelihood of experiencing upward wealth mobility for Black and white families, based on their initial employment status. It can be seen that for both Black and white business owners, entrepreneurship is associated with a higher likelihood of experiencing upward mobility.

**Table 6:** Logit regressions predicting family transitions in the wealth distribution based on employment status at the start of each 4-year period, entrepreneurs and workers only. Dependent variable: family rises into the top third from below the top third

	Model 1			Model 2		
	Coefficient	*Z* score		Coefficient	*Z* score	
Status at start						
Entrepreneur	0.3934	2.9	**	0.3946	2.8	**
Black reference person	-0.3134	-2.9	**	-0.3126	-2.8	**
Status at start × Race						
Black entrepreneur				-0.0148	0.0	
Education						
Less than high school	-0.4040	-2.8	**	-0.4040	-2.8	**
Some college	0.3160	3.1	**	0.3160	3.1	**
Bachelor's degree	0.6019	5.6	***	0.6019	5.6	***
More than bachelor's degree	0.9489	7.5	***	0.9490	7.5	***
Age (in years)						
Under 35	-0.3186	-3.8	***	-0.3185	-3.7	***
Over 54	-0.0433	-0.4		-0.0433	-0.4	
Household type						
Single male	-0.2323	-2.2		-0.2323	-2.2	
Single female	-0.7972	-6.7	***	-0.7972	-6.7	***
Number of children under 18	-0.1572	-4.2	***	-0.1572	-4.2	***
Own home	0.7813	8.5	***	0.7813	8.4	***
Health good or better	0.2461	1.7	☨	0.2460	1.7	☨
Received gift or inheritance	0.6526	4.7	***	0.6525	4.7	***
Middle third wealth at start	0.8594	10.4	***	0.8594	10.4	***
Period						
2001–2005	-0.4350	-4.0	***	-0.4350	-4.0	***
2005–2009	-0.3108	-3.0	**	-0.3107	-3.0	**
2009–2013	-0.2048	-2.0	*	-0.2048	-2.0	*
Constant	-2.3887	-12.2	***	-2.3889	-12.2	***

*N*=10,417. Regression includes only entrepreneurs and workers at the start of the period. Robust standard errors clustered by family are used. Reference categories are, respectively, worker, white reference person, Black worker, high school degree, ages 35 to 54, married, no children under 18, does not own home, health worse than “good,” did not receive gift or inheritance, top or bottom third wealth at start, and 2013–2017. Transitions are measured across 4-year periods from 2001 to 2017. Estimated using PSID data (2020)

☨*p*<0.1, *p<0.05, **p<0.01, ***p<0.001

Table 7 shows comparable models predicting downward economic mobility. In Model 1, the coefficient for entrepreneurship is positive, but not significant, indicating that reference persons who are entrepreneurs at a given time point are not significantly more likely to experience downward wealth mobility over the next 4 years, compared to workers. In Model 2, the interaction term between race and entrepreneurship is positive and significant at the 99% level. This coefficient suggests that being an entrepreneur (versus being a worker) is associated with a higher risk of downward mobility for Black people than for white people with similar socio-economic characteristics.

**Table 7:** Logit regressions predicting family transitions in the wealth distribution based on employment status at the start of each 4-year period, entrepreneurs and workers only. Dependent variable: family falls into the bottom third from above the bottom third

	Model 1			Model 2		
	Coefficient	*Z* score		Coefficient	*Z* score	
Status at start						
Entrepreneur	0.1323	1.1		0.0241	0.2	
Black reference person	0.3766	3.6	***	0.3063	2.9	**
Status at start × Race						
Black entrepreneur				0.9361	2.8	**
Education						
Less than high school	0.2277	1.8	☨	0.2246	1.8	☨
Some college	0.0231	0.2		0.0167	0.2	
Bachelor's degree	-0.3992	-3.4	***	-0.4077	-3.5	***
More than bachelor's degree	-0.4540	-2.9	**	-0.4665	-2.9	**
Age (in years)						
Under 35	0.2354	2.6	*	0.2348	2.5	*
Over 54	-0.2665	-2.3	*	-0.2606	-2.2	*
Household type						
Single male	0.2818	2.7	**	0.2711	2.6	*
Single female	0.5867	5.7	***	0.5890	5.7	***
Number of children under 18	0.1475	4.1	***	0.1453	4.1	***
Own home	-0.6014	-6.1	***	-0.6036	-6.2	***
Health good or better	-0.4641	-3.5	***	-0.4506	-3.5	***
Received gift or inheritance	-0.5109	-3.4	***	-0.5042	-3.3	***
Middle third wealth at start	1.2316	12.1	***	1.2287	12.1	***
Period						
2001–2005	0.1631	1.5		0.1661	1.5	
2005–2009	0.1159	1.1		0.1123	1.0	
2009–2013	-0.1404	-1.3		-0.1394	-1.3	
Constant	-2.0733	-10.0	***	-2.0603	-10.0	***

Note: *N*=11,187. Regression includes only entrepreneurs and workers at the start of the period. Robust standard errors clustered by family are used. Reference categories are, respectively, worker, white reference person, Black worker, high school degree, ages 35 to 54, married, no children under 18, does not own home, health worse than “good,” did not receive gift or inheritance, top or bottom third wealth at start, and 2013–2017. Transitions are measured across 4-year periods from 2001 to 2017. Estimated using PSID data (2020)

☨*p*<0.1, *p<0.05, **p<0.01, ***p<0.001

**Fig. 4:**
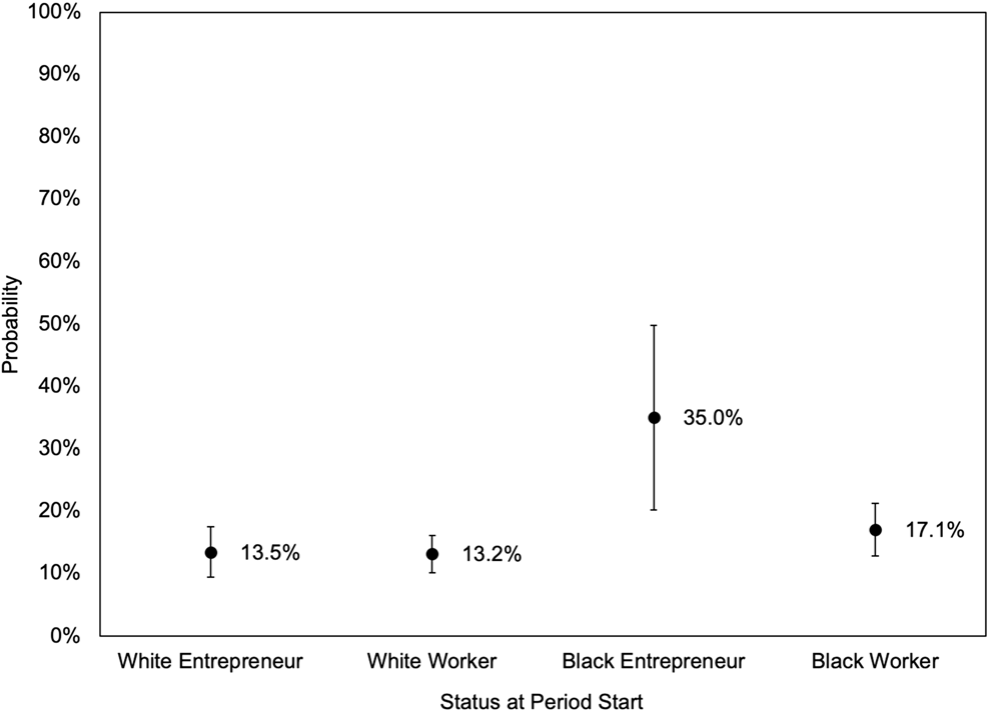
Predicted probability of falling into the bottom wealth tercile from above the bottom wealth tercile, by race and status at start. *Note:* *Error bars represent 95% confidence intervals. Predictions derived from Model 2 in Table* *7*. *Other variables held at the following values: high school degree, age 35 to 54, married, no children under 18, owns home, health better than* “*good,*” *did not receive gift or inheritance, middle third wealth at start, and 2013*–*2017. Transitions are measured across 4-year periods from 2001* *to* *2017*

The magnitude of this effect is shown in Fig. 4. Among white people, entrepreneurs have a similar likelihood of experiencing downward mobility over the next 4 years as those who were workers the entire time. Among Black people however, entrepreneurs appear far more likely to experience downward wealth mobility than workers. These findings comport with the other results presented in Tables 3 and 5, showing that unsuccessful entrepreneurs are more likely to experience downward wealth mobility and that Black entrepreneurs are more likely to be unsuccessful.

## Discussion

Despite the limitations noted above, these results have implications for research and policy on the racial wealth gap. Firstly, our results echo earlier findings that Black-owned businesses tend to end sooner than their white-owned counterparts. Secondly, our analyses make clear that entrepreneurship is not a sure ticket to upward economic mobility. As long as a business continues to operate, entrepreneurs are indeed more likely to experience upward wealth mobility compared to comparable workers. However, when a business closes, entrepreneurs face a higher likelihood of experiencing downward wealth mobility compared to workers, suggesting that business failure poses a serious risk to the wealth position of aspiring entrepreneurs, regardless of race.

Neither of these two findings should be controversial, or even particularly surprising, but in combination they have important, but thus far unappreciated, implications for debates about the role of entrepreneurship in the racial wealth gap. In particular, they suggest that merely increasing the rate at which Black people start businesses may actually *exacerbate* the racial wealth gap.^10^ This dynamic is clearly illustrated by Figs. 3 and 4, which show that a Black entrepreneur who begins in the middle wealth tercile has roughly a 39% probability of rising into the top tercile over the next 4 years, and a 35% probability of falling into the bottom tercile. In contrast, a comparable white entrepreneur who begins in the middle tercile has a 47% probability of rising into the top tercile over the next 4 years, and only a 14% probability of falling into the bottom tercile. Our analyses suggest that these dramatic differences are primarily due to the differences in success rates for Black and white entrepreneurs.

Indeed, the overall claim that racial economic disparities could be reduced by simply increasing the rate at which Black people start businesses are belied by the fact that, during the past few decades, minority group members were *already* more likely to start businesses compared to white people (Fairlie, 2012; Köllinger & Minniti, 2006). The racial wealth gap, however, has continued to increase during this period (Shapiro et al., 2013), and our analyses suggests that the failure of many of these businesses may actually be contributing to widening of racial wealth disparities in the US. Rather, this work suggests that the rate at which Black-owned businesses succeed is the most important factor for determining the effect of entrepreneurship on the racial wealth gap. Increasing this rate would both increase the proportion of Black entrepreneurs who experience the upward wealth mobility shown in Fig. 3 as well as decrease the proportion who experience the downward mobility as shown in Fig. 4, and thus have a profound impact on the overall wealth position of current and aspiring Black entrepreneurs.^11^

Fortunately, research has already identified many of the key drivers of business failure among Black entrepreneurs. Chief among these, as noted above, is a lack of access to quality credit and financing (Fairlie & Robb, 2008). Thus, increasing the opportunities for Black entrepreneurs to access fair and affordable financing has great potential to substantially reduce the racial wealth gap. This approach avoids the potential negative consequences associated with promoting higher rates of Black entrepreneurship, which are already fairly high in any case.^12^

## Conclusion

Entrepreneurship is sometimes framed as a ticket to economic prosperity, but it should perhaps be thought of as a highly leveraged bet. Those who win the bet may reap a huge economic payoff, but those who lose may face financial ruin. Our findings suggest that, when playing this game, white and Black entrepreneurs do not have the same odds of winning. This simple fact implies that convincing more Black people to make a dangerous bet when they have lower odds of winning than white people is not a promising approach to reducing the racial wealth gap. Changing the odds for Black entrepreneurs who have already placed their bet, however, appears to be a much more attractive option.

## Supplementary Information

ESM 1 (DOCX 62.8 kb)

## Footnotes

1 Toney and Price (2020) also explore whether entrepreneurship could alleviate racial disparities in emotional “well-being,” but do not explore its impact on economic inequality or the racial wealth gap.

2 The PSID uses the phrase “reference person” in 2017 to replace “head of household” used in previous years. “Head” was formerly used to refer to the husband in a heterosexual married couple, or to a single adult of either sex.

3 Because the “reference person” of a PSID family can change from one wave to the next, we include only families where the reference person represents the same individual in both time points.

4 Wealth is based on the PSID's constructed wealth variable. Non-equity wealth is comprised of the value of seven asset types (farm or business, cash, other real estate, stocks, vehicles, other assets, and IRAs or annuity), net the value of debts (from farm or business, other real estate, credit card, student loans, medical, legal, family loans, and other debts). Equity wealth includes the above plus home value less mortgages.

5 To explore the implications of excluding home equity, we ran an alternate version of the analysis discussed in this paper using the full wealth variable. Results of this sensitivity analysis are discussed in footnote 11 and in the supplementary appendix.

6 See, e.g., Fairlie and Robb (2008), “Relatively low levels of wealth among blacks and the existence of liquidity constraints in U.S. financial markets may limit the ability of black entrepreneurs to raise the optimal levels of capital needed to start businesses,” (p. 107) and Boston (1999), “Atlanta’s experience proves that entrepreneurship is not dependent upon the culture of a people, and developing black owned businesses can be accelerated if racial barriers are reduced” (p. 3).

7 We adopt these definitions to align our research with previous studies; however, we acknowledge the limitations of referring to this group as “unsuccessful entrepreneurs.” Individuals who transition from entrepreneurship to working or to unemployment may have a variety of reasons for which they do so. Nonetheless, this shorthand remains a convenient and probable description of the employment transition.

8 The category “other” captures all other employment transitions between working, unemployed, retired, and being an entrepreneur.

9 Future analyses should explore the impact of entrepreneurship on *absolute* wealth change, as demonstrated by Meschede et al. (2016).

10 One potential criticism of our findings is that individuals who transition from entrepreneur to retired, categorized here as “other”, may do so as a result of business success (i.e., selling a lucrative business). In this case, we would expect to see the models that disregard status at end (Tables 6 and 7) to illustrate a different picture than the models that include status at the end of the period (Tables 4 and 5). We by and large do not find disparate results between these models, suggesting that the data are not heavily biased by wealthy investors who transition from entrepreneur to retired.

11 As mentioned in footnote 5, we also explored alternative models that analyzed total (as opposed to non-equity) wealth. These models (reported in Tables A1–A6 in the electronic supplementary appendix) produced similar results to those reported here, suggesting that our decisions to analyze non-equity wealth did not substantively impact our conclusions. One of the only substantive changes was in regard to the interaction term between Black and unsuccessful entrepreneur in the model of falling into the bottom third of the income distribution. In the reported model (Model 2 in Table 5), this coefficient is positive but non-significant. In the sensitivity analysis (Table A4) it is positive and significant at the .05 level, implying that Black unsuccessful entrepreneurs do have a greater likelihood of experiencing downward mobility than white unsuccessful entrepreneurs. Although this significant result should be treated as tentative since it is not robust to alternative specifications of the model, it suggests that, notwithstanding Black entrepreneurs’ greater likelihood of experiencing business failure, the economic cost of such failures may actually be more severe for Black entrepreneurs than for comparable white entrepreneurs.

12 Some recent policy proposals have already promoted this approach. For example, in 2019, Democratic Presidential candidate Elizabeth Warren proposed a plan that would offer $7 billion in grants to entrepreneurs who are eligible for the Small Business Administration’s existing 8(a) business development program and have less than $100,000 in household wealth (Warren, 2019). Warren explicitly framed this program as targeting the racial wealth gap, and because it targets existing entrepreneurs, and addresses one of the biggest barriers to Black entrepreneurs, it is broadly in line with past and current research.

## References

[CR1] Abraham KG, Hershbein B, Houseman S. Independent contract and informal work: preliminary evidence on developing better measures in household surveys. Atlanta, Georgia: Meeting of the Allied Social Science Associations; 2018.

[CR2] Ahn T. Racial differences in self-employment exits. Small Bus Econ. 2011;36(1):169–86.

[CR3] Asiedu E, Freeman JA, Nti-Addae A. Access to credit by small businesses: How relevant are race, ethnicity, and gender. Am Econ Rev. 2012;102(3):532–7.

[CR4] Bates T. The changing nature of minority business: A comparative analysis of Asian, nonminority, and black-owned businesses. Rev Black Polit Econ. 1989;18(2):25–42.

[CR5] Bates T, Robb A. Has the Community Reinvestment Act increased loan availability among small businesses operating in minority neighbourhoods? Urban Stud. 2015;52(9):1702–21.

[CR6] Bates T, Robb A. Impacts of owner race and geographic context on access to small-business financing. Econ Dev Q. 2016;30(2):159–70.

[CR7] Blanchard L, Zhao B, Yinger J. Do lenders discriminate against minority and woman entrepreneurs? J Urban Econ. 2008;63(2):467–97.

[CR8] Blanchflower DG, Levine PB, Zimmerman DJ. Discrimination in the small-business credit market. Rev Econ Stat. 2003;85(4):930–43.

[CR9] Bone SA, Christensen GL, Williams JD. Rejected, shackled, and alone: the impact of systemic restricted choice on minority consumers’ construction of self. J Consum Res. 2014;41(1):451–74.

[CR10] Bone SA, Christensen GL, Williams JD, Adams S, Lederer A, Lubin PC. Detecting discrimination in small business lending. Management Faculty Publication, Paper 366. 2017.

[CR11] Boston TD. Affirmative action and black entrepreneurship: Routledge; 1999.

[CR12] Bradford WD. The “Myth” that black entrepreneurship can reduce the gap in wealth between black and white families. Econ Dev Q. 2014;28(3):254–69.

[CR13] Butler JS. Entrepreneurship and self-help among black Americans: State University of New York Press; 1991.

[CR14] Cope J. Entrepreneurial learning from failure: An interpretative phenomenological analysis. J Bus Ventur. 2011;26:604–23.

[CR15] Fairlie RW. Open For business: How immigrants are driving small business creation in the United States: The Partnership for a New American Economy; 2012. http://research.newamericaneconomy.org/wp-content/uploads/2013/07/openforbusiness.pdf.

[CR16] Fairlie RW. The impact of COVID-19 on small business owners: Evidence from the first three months after widespread social-distancing restrictions. J Econ Manag Strateg. 2020;29(4):727–40. 10.1111/jems.12400. 10.1111/jems.12400 PMC7461311 32904856

[CR17] Fairlie RW, Robb AM. Race and entrepreneurial success: black-, asian-, and white-owned businesses in the United States: The MIT Press; 2008.

[CR18] Gittleman M, Wolff EN. Racial differences in patterns of wealth accumulation. J Hum Resour. 2004;39(1):193–227.

[CR19] Hamilton D, Darity W Jr. Can ‘baby bonds’ eliminate the racial wealth gap in putative post-racial America? Rev Black Polit Econ. 2010;37(1):207–16.

[CR20] Headd B. Redefining business success: Distinguishing between closure and failure. Small Bus Econ. 2003;21:51–61.

[CR21] Köllinger P, Minniti M. Not for lack of trying: American Entrepreneurship in Black and White. Small Bus Econ. 2006;27:59–79.

[CR22] Lubin PC. Protecting main street: measuring customer experience in financial services for business and public policy: Routledge; 2011.

[CR23] Meschede T, Thomas H, Mann A, Stagg A, Shapiro T. Wealth mobility of families raising children in the twenty-first century. Race Soc Probl. 2016;8(1):77–92.

[CR24] Oliver ML, Shapiro T. Black wealth/white wealth: a new perspective on racial inequality: Oxford University Press; 2004.

[CR25] Panel Study of Income Dynamics, public use dataset. Produced and distributed by the survey research center. Institute for Social Research. Ann Arbor, MI: University of Michigan; 2020.

[CR26] Phillips BD, Kirchhoff BA. Formation, growth and survival; small firm dynamics in the U.S. Economy. Small Bus Econ. 1989;1(1):65–74.

[CR27] Piketty T. Capital in the twenty-first century (A. Goldhammer, Trans.). The Belknap Press of Harvard University Press. 2014.

[CR28] Quadrini V. The importance of entrepreneurship for wealth concentration and mobility. Rev Income Wealth. 1999;45(1):1–19.

[CR29] Shapiro T. The hidden cost of being african american: Oxford University Press; 2004.

[CR30] Shapiro T, Meschede T, Osoro S. The roots of the widening racial wealth gap: explaining the black-white economic divide: Institute on Assets and Social Policy; 2013. http://iasp.brandeis.edu/pdfs/Author/shapiro-thomas-m/racialwealthgapbrief.pdf.

[CR31] Sherraden M. Assets and the Poor Inc: M.E. Sharpe; 1991.

[CR32] Sullivan L, Meschede T, Dietrich L, Shapiro T, Huelsman M, Draut T. Less debt, more equity: lowering student debt while closing the black-white wealth gap. Institute on Assets and Social Policy & Demos; 2015.

[CR33] Sullivan L, Meschede T, Shapiro T, Escobar MF. Misdirected investments: how the mortgage interest deduction drives inequality and the racial wealth gap: Institute on Assets and Social Policy & National Low Income Housing Coalition; 2017.

[CR34] Toney SL, Price GN. Can black entrepreneurship reduce black-white inequality in the United States? J Econ Race Policy. 2020. 10.1007/s41996-020-00065-y.

[CR35] Turner MA, Freiberg F, Godfrey E, Herbig C, Levy DK, Smith RR. All other things being equal: a paired testing study of mortgage lending institutions. The Urban Institute. http://webarchive.urban.org/UploadedPDF/1000504_All_Other_Things_Being_Equal.pdf.

[CR36] Ucbasaran D, Shepherd DA, Lockett A, Lyon SJ. Life after business failure: the process and consequences of business failure for entrepreneurs. J Manag. 2013;39(1):163–202.

[CR37] Warren E. Leveling the playing field for entrepreneurs. 2019. https://medium.com/@teamwarren/leveling-the-playing-field-for-entrepreneurs-2a585aa2b6d7.

