# Supplementary material for: Entrepreneurship and the Racial Wealth Gap: The Impact of Entrepreneurial Success or Failure on the Wealth Mobility of Black and White Families
Source: J Econ Race Policy. 2021 Feb 22;4(3):183–95. doi: 10.1007/s41996-021-00081-6 (PMC7897735; doi:10.1007/s41996-021-00081-6)
Supplement: Supplementary file 1 — (DOCX 62.8 kb) [file 41996_2021_81_MOESM1_ESM.docx]

**Electronic Supplementary Appendix to “Entrepreneurship and the Racial Wealth Gap: The Impact of Entrepreneurial Success or Failure on the Wealth Mobility of Black and White Families”**

Teresa Kroeger & Graham Wright

Main Paper: <http://dx.doi.org/10.1007/s41996-021-00081-6>

To explore the implications of excluding home equity from wealth, we ran an alternate version of the analysis discussed in the main paper using the full equity wealth variable. These models produced similar results to those reported in the main text of the paper, suggesting that our decisions to analyze non-equity wealth did not substantively impact our conclusions. Results of this sensitivity analysis are shown below.

Our analyses utilize the PSID’s constructed wealth variable. Non-equity wealth is comprised of the value of seven asset types (farm or business, cash, other real estate, stocks, vehicles, other assets, and IRAs or annuity), net the value of debts (from farm or business, other real estate, credit card, student loans, medical, legal, family loans, and other debts). Equity wealth includes the above plus home value less mortgages. All tables presented in this appendix use equity wealth.

**Table A1** Family traits in combined periods 2001–2005, 2005–2009, 2009–2013, and 2013–2017, using wealth with home equity

|  | All families | White families | Black families |  |
| --- | --- | --- | --- | --- |
| Middle third wealth at start | 0.296 | 0.281 | 0.374 | *** |
| Top two thirds at start | 0.746 | 0.790 | 0.518 | *** |
| Top two thirds at end | 0.743 | 0.788 | 0.510 | *** |
| Average wealth at start ($) | 347,087 | 398,619 | 77,893 | *** |
| Standard deviation | 1,679,749 | 1,819,931 | 415,824 |  |
| Average wealth at end ($) | 415,532 | 478,767 | 85,201 | *** |
| Standard deviation | 1,881,998 | 2,039,751 | 425,082 |  |
| N | 22,958 | 14,271 | 8,687 |  |
| ☨p<0.1, * p<0.05, ** p<0.01, *** p<0.001 | | | | |
| Note: Significance values from design-adjusted chi-square and *t* tests. Wealth is in nominal dollars and includes home equity. Estimated using PSID data (2020). | | | | |

**Table A2** Logit regression predicting successful entrepreneurship, using wealth with home equity. Dependent variable: entrepreneur at the start and end of the period

|  | **Model 1** | | |
| --- | --- | --- | --- |
|  | Coefficient | Z score | |
| Black reference person | -0.8034 | -3.0 | ** |
| Education |  |  |  |
| Less than high school | -0.2810 | -1.1 |  |
| Some college | -0.1997 | -1.1 |  |
| Bachelor's degree | -0.0976 | -0.5 |  |
| More than bachelor's degree | 0.2095 | 0.9 |  |
| Age (in years) |  |  |  |
| Under 35 | -0.2557 | -1.5 |  |
| Over 54 | -0.0557 | -0.3 |  |
| Household type |  |  |  |
| Single male | 0.1207 | 0.6 |  |
| Single female | -0.1386 | -0.5 |  |
| Number of children under 18 | -0.1165 | -1.8 | ☨ |
| Own home | 0.0843 | 0.4 |  |
| Health good or better | 0.4097 | 1.6 |  |
| Received gift or inheritance | -0.1245 | -0.7 |  |
| Wealth tercile |  |  |  |
| Bottom third wealth at start | -1.0963 | -4.9 | *** |
| Middle third wealth at start | -0.6468 | -3.9 | *** |
| Period |  |  |  |
| 2001-2005 | -0.1029 | -0.6 |  |
| 2005-2009 | 0.0039 | 0.0 |  |
| 2009-2013 | -0.0491 | -0.3 |  |
| Constant | 0.7649 | 2.1 | * |
| ☨p<0.1, * p<0.05, ** p<0.01, *** p<0.001 | | | |
| Note: N=1,810. Wealth includes home equity. Regression includes only entrepreneurs at the start of the period. Robust standard errors clustered by family are used. Reference categories are, respectively, white head of household, high school degree, ages 35 to 54, married, no children under 18, does not own home, health worse than “good,” did not receive gift or inheritance, top or bottom third wealth at start, and 2013–2017. Transitions are measured across 4-year periods from 2001-2017. Estimated using PSID data (2020). | | | |

**Table A3** Logit regressions predicting family transitions in the wealth distribution based on employment transition, using wealth with home equity. Dependent variable: family rises into the top third from below the top third

|  | **Model 1** | | | **Model 2** | | |
| --- | --- | --- | --- | --- | --- | --- |
|  | Coefficient | Z score | | Coefficient | Z score | |
| Employment transition |  |  |  |  |  |  |
| Successful entrepreneur | 0.7995 | 4.5 | *** | 0.7462 | 4.0 | *** |
| Unsuccessful entrepreneur | 0.1625 | 0.7 |  | 0.2039 | 0.9 |  |
| Otherª | 0.1385 | 1.4 |  | 0.1062 | 1.0 |  |
| Black reference person | -0.3048 | -2.7 | ** | -0.3943 | -2.7 | ** |
| Employment transition x Race |  |  |  |  |  |  |
| Black successful entrepreneur |  |  |  | 0.7710 | 1.5 |  |
| Black unsuccessful entrepreneur |  |  |  | -0.5559 | -1.0 |  |
| Black other |  |  |  | 0.1764 | 0.8 |  |
| Education |  |  |  |  |  |  |
| Less than high school | -0.6158 | -4.3 | *** | -0.6170 | -4.3 | *** |
| Some college | 0.3966 | 3.8 | *** | 0.3972 | 3.8 | *** |
| Bachelor's degree | 0.7327 | 6.7 | *** | 0.7324 | 6.7 | *** |
| More than bachelor's degree | 0.9441 | 7.2 | *** | 0.9395 | 7.2 | *** |
| Age (in years) |  |  |  |  |  |  |
| Under 35 | -0.3373 | -3.9 | *** | -0.3389 | -3.9 | *** |
| Over 54 | 0.0358 | 0.3 |  | 0.0382 | 0.3 |  |
| Household type |  |  |  |  |  |  |
| Single male | -0.3188 | -3.0 | ** | -0.3177 | -3.0 | ** |
| Single female | -0.7585 | -6.8 | *** | -0.7564 | -6.8 | *** |
| Number of children under 18 | -0.1161 | -3.2 | ** | -0.1168 | -3.2 | ** |
| Own home | 0.5463 | 5.8 | *** | 0.5430 | 5.7 | *** |
| Health good or better | 0.4798 | 3.5 | *** | 0.4807 | 3.5 | *** |
| Received gift or inheritance | 0.6693 | 5.5 | *** | 0.6674 | 5.5 | *** |
| Middle third wealth at start | 1.0867 | 10.4 | *** | 1.0881 | 10.4 | *** |
| Period |  |  |  |  |  |  |
| 2001-2005 | -0.2583 | -2.5 | * | -0.2604 | -2.5 | * |
| 2005-2009 | -0.1408 | -1.4 |  | -0.1416 | -1.4 |  |
| 2009-2013 | -0.1781 | -1.7 | ☨ | -0.1776 | -1.7 | ☨ |
| Constant | -3.1875 | -16.2 | *** | -3.1741 | -16.0 | *** |
| ☨p<0.1, * p<0.05, ** p<0.01, *** p<0.001 | | | |  | | |
| ª Excludes stayed worker |  |  |  |  |  |  |
| Note: N=14,683. Wealth includes home equity. Robust standard errors clustered by family are used. Reference categories are, respectively, stayed worker, white reference person, Black stayed worker, high school degree, ages 35 to 54, married, no children under 18, does not own home, health worse than “good,” did not receive gift or inheritance, top or bottom third wealth at start, and 2013–2017. Transitions are measured across 4-year periods from 2001-2017. Estimated using PSID data (2020). | | | | | | |

**Table A4** Logit regressions predicting family transitions in the wealth distribution based on employment transition, using wealth with home equity. Dependent variable: family falls into the bottom third from above the bottom third

|  | **Model 1** | | | **Model 2** | | |
| --- | --- | --- | --- | --- | --- | --- |
|  | Coefficient | Z score | | Coefficient | Z score | |
| Employment transition |  |  |  |  |  |  |
| Successful entrepreneur | -0.1973 | -1.0 |  | -0.2502 | -1.2 |  |
| Unsuccessful entrepreneur | 0.5854 | 2.9 | ** | 0.4450 | 1.9 | ☨ |
| Otherª | 0.4094 | 4.5 | *** | 0.4159 | 4.1 | *** |
| Black reference person | 0.2433 | 2.4 | * | 0.2198 | 1.7 |  |
| Employment transition x Race |  |  |  |  |  |  |
| Black successful entrepreneur |  |  |  | 0.7571 | 1.5 |  |
| Black unsuccessful entrepreneur |  |  |  | 0.9658 | 2.1 | * |
| Black other |  |  |  | -0.0217 | -0.1 |  |
| Education |  |  |  |  |  |  |
| Less than high school | 0.2557 | 2.3 | * | 0.2554 | 2.3 | * |
| Some college | -0.0731 | -0.7 |  | -0.0772 | -0.8 |  |
| Bachelor's degree | -0.5441 | -4.3 | *** | -0.5493 | -4.3 | *** |
| More than bachelor's degree | -0.7872 | -4.8 | *** | -0.7924 | -4.9 | *** |
| Age (in years) |  |  |  |  |  |  |
| Under 35 | 0.1292 | 1.4 |  | 0.1283 | 1.4 |  |
| Over 54 | -0.5982 | -5.4 | *** | -0.6006 | -5.5 | *** |
| Household type |  |  |  |  |  |  |
| Single male | 0.2963 | 2.7 | ** | 0.2944 | 2.7 | ** |
| Single female | 0.7532 | 7.7 | *** | 0.7535 | 7.8 | *** |
| Number of children under 18 | 0.1673 | 4.6 | *** | 0.1669 | 4.6 | *** |
| Own home | -1.2231 | -13.3 | *** | -1.2227 | -13.1 | *** |
| Health good or better | -0.5875 | -5.6 | *** | -0.5852 | -5.6 | *** |
| Received gift or inheritance | -0.4029 | -2.8 | ** | -0.3946 | -2.7 | ** |
| Middle third wealth at start | 1.4936 | 13.8 | *** | 1.4955 | 13.8 | *** |
| Period |  |  |  |  |  |  |
| 2001-2005 | 0.1985 | 1.8 | ☨ | 0.2026 | 1.9 | ☨ |
| 2005-2009 | 0.1747 | 1.6 |  | 0.1764 | 1.7 | ☨ |
| 2009-2013 | -0.0890 | -0.9 |  | -0.0868 | -0.9 |  |
| Constant | -2.0022 | -10.4 | *** | -1.9999 | -10.3 | *** |
| ☨p<0.1, * p<0.05, ** p<0.01, *** p<0.001 | | | |  | | |
| ª Excludes stayed worker |  |  |  |  |  |  |
| Note: N=14,939. Wealth includes home equity. Robust standard errors clustered by family are used. Reference categories are, respectively, stayed worker, white reference person, Black stayed worker, high school degree, ages 35 to 54, married, no children under 18, does not own home, health worse than “good,” did not receive gift or inheritance, top or bottom third wealth at start, and 2013–2017. Transitions are measured across 4-year periods from 2001-2017. Estimated using PSID data (2020). | | | | | | |

**Table A5** Logit regressions predicting family transitions in the wealth distribution based on employment status at the start of each 4-year period, entrepreneurs and workers only, using wealth with home equity. Dependent variable: family rises into the top third from below the top third

|  | **Model 1** | | | **Model 2** | | |
| --- | --- | --- | --- | --- | --- | --- |
|  | Coefficient | Z score | | Coefficient | Z score | |
| Status at start |  |  |  |  |  |  |
| Entrepreneur | 0.4841 | 3.5 | *** | 0.4792 | 3.3 | *** |
| Black reference person | -0.3541 | -2.8 | ** | -0.3582 | -2.7 | ** |
| Status at start x Race |  |  |  |  |  |  |
| Black entrepreneur |  |  |  | 0.0621 | 0.2 |  |
| Education |  |  |  |  |  |  |
| Less than high school | -0.5643 | -3.4 | *** | -0.5644 | -3.4 | *** |
| Some college | 0.3826 | 3.4 | *** | 0.3823 | 3.4 | *** |
| Bachelor's degree | 0.7557 | 6.7 | *** | 0.7552 | 6.7 | *** |
| More than bachelor's degree | 0.9598 | 7.1 | *** | 0.9596 | 7.1 | *** |
| Age (in years) |  |  |  |  |  |  |
| Under 35 | -0.3414 | -3.8 | *** | -0.3417 | -3.8 | *** |
| Over 54 | 0.1320 | 1.0 |  | 0.1321 | 1.0 |  |
| Household type |  |  |  |  |  |  |
| Single male | -0.2638 | -2.4 |  | -0.2638 | -2.4 |  |
| Single female | -0.7871 | -6.3 | *** | -0.7870 | -6.3 | *** |
| Number of children under 18 | -0.1386 | -3.6 | *** | -0.1387 | -3.6 | *** |
| Own home | 0.5586 | 5.5 | *** | 0.5585 | 5.5 | *** |
| Health good or better | 0.3359 | 2.0 | * | 0.3360 | 2.0 | * |
| Received gift or inheritance | 0.7161 | 5.5 | *** | 0.7162 | 5.5 | *** |
| Middle third wealth at start | 1.1355 | 10.3 | *** | 1.1357 | 10.3 | *** |
| Period |  |  |  |  |  |  |
| 2001-2005 | -0.2536 | -2.2 | * | -0.2534 | -2.2 | * |
| 2005-2009 | -0.2660 | -2.4 | * | -0.2660 | -2.4 | * |
| 2009-2013 | -0.2209 | -1.9 | ☨ | -0.2209 | -1.9 | ☨ |
| Constant | -2.9855 | -13.1 | *** | -2.9847 | -13.1 | *** |
| ☨p<0.1, * p<0.05, ** p<0.01, *** p<0.001 | | | | | | |
| Note: N=10,417. Wealth includes home equity. Regression includes only entrepreneurs and workers at the start of the period. Robust standard errors clustered by family are used. Reference categories are, respectively, worker, white reference person, Black worker, high school degree, ages 35 to 54, married, no children under 18, does not own home, health worse than “good,” did not receive gift or inheritance, top or bottom third wealth at start, and 2013–2017. Transitions are measured across 4-year periods from 2001-2017. Estimated using PSID data (2020). | | | | | | |

**Table A6** Logit regressions predicting family transitions in the wealth distribution based on employment status at the start of each 4-year period, entrepreneurs and workers only, using wealth with home equity. Dependent variable: family falls into the bottom third from above the bottom third

|  | **Model 1** | | | **Model 2** | | |
| --- | --- | --- | --- | --- | --- | --- |
|  | Coefficient | Z score | | Coefficient | Z score | |
| Status at start |  |  |  |  |  |  |
| Entrepreneur | 0.0500 | 0.4 |  | -0.0568 | -0.4 |  |
| Black reference person | 0.3260 | 3.0 | ** | 0.2644 | 2.3 | * |
| Status at start x Race |  |  |  |  |  |  |
| Black entrepreneur |  |  |  | 0.9485 | 2.8 | ** |
| Education |  |  |  |  |  |  |
| Less than high school | 0.4135 | 3.1 | ** | 0.4096 | 3.0 | ** |
| Some college | -0.0022 | 0.0 |  | -0.0109 | -0.1 |  |
| Bachelor's degree | -0.5482 | -4.1 | *** | -0.5593 | -4.2 | *** |
| More than bachelor's degree | -0.8186 | -4.5 | *** | -0.8288 | -4.6 | *** |
| Age (in years) |  |  |  |  |  |  |
| Under 35 | 0.1699 | 1.7 |  | 0.1666 | 1.7 | ☨ |
| Over 54 | -0.3131 | -2.3 | * | -0.3108 | -2.3 | * |
| Household type |  |  |  |  |  |  |
| Single male | 0.3270 | 2.7 | ** | 0.3214 | 2.7 | ** |
| Single female | 0.6057 | 5.2 | *** | 0.6088 | 5.2 | *** |
| Number of children under 18 | 0.1913 | 5.0 | *** | 0.1895 | 4.9 | *** |
| Own home | -1.1244 | -10.6 | *** | -1.1293 | -10.6 | *** |
| Health good or better | -0.6892 | -5.0 | *** | -0.6812 | -5.0 | *** |
| Received gift or inheritance | -0.3158 | -1.9 | ☨ | -0.3084 | -1.8 | ☨ |
| Middle third wealth at start | 1.4915 | 11.7 | *** | 1.4888 | 11.7 | *** |
| Period |  |  |  |  |  |  |
| 2001-2005 | 0.2983 | 2.4 | * | 0.3038 | 2.5 | * |
| 2005-2009 | 0.2776 | 2.2 | * | 0.2807 | 2.3 | * |
| 2009-2013 | 0.0115 | 0.1 |  | 0.0145 | 0.1 |  |
| Constant | -2.0484 | -8.9 | *** | -2.0321 | -8.8 | *** |
| ☨p<0.1, * p<0.05, ** p<0.01, *** p<0.001 | | | | | | |
| Note: N=11,187. Wealth includes home equity. Regression includes only entrepreneurs and workers at the start of the period. Robust standard errors clustered by family are used. Reference categories are, respectively, worker, white reference person, Black worker, high school degree, ages 35 to 54, married, no children under 18, does not own home, health worse than “good,” did not receive gift or inheritance, top or bottom third wealth at start, and 2013–2017. Transitions are measured across 4-year periods from 2001-2017. Estimated using PSID data (2020). | | | | | | |
